# Transverse myelitis after anti‐CD19 directed CAR T cell therapy for relapsed large B cell lymphoma

**DOI:** 10.1002/jha2.286

**Published:** 2021-12-06

**Authors:** Semira Sheikh, Sepideh Mokhtari, Jeffrey A. Silverman, Kayla Reid, Rawan Faramand, Marco L. Davila, Norman Franke, Frederick L. Locke, Michael D. Jain, Daniel Wong, John G. Kuruvilla

**Affiliations:** ^1^ Division of Medical Oncology and Hematology University Health Network Toronto Canada; ^2^ Department of Neuro‐Oncology Moffitt Cancer Center Tampa Florida USA; ^3^ Department of Hematology North York General Hospital Toronto Canada; ^4^ Department of Hematology Moffitt Cancer Center Tampa Florida USA; ^5^ Department of Malignant Hematology Moffitt Cancer Center Tampa Florida USA; ^6^ Department of Blood & Marrow Transplant and Cellular Immunotherapy Moffitt Cancer Center Tampa Florida USA; ^7^ Division of Medical Oncology and Hematology Princess Margaret Hospital Toronto Canada; ^8^ Department of Neurology North York General Hospital Toronto Canada

**Keywords:** CAR T cell therapy, ICANS, neurotoxicity, transverse myelitis

Chimeric antigen receptor (CAR) T cell therapy has transformed the care of patients with relapsed/refractory large B cell lymphoma and is associated with unique toxicities, in particular cytokine release syndrome (CRS) and neurotoxicity/immune effector cell‐associated encephalopathy syndrome (ICANS) [1, 2, 3]. The pathophysiology of neurotoxicity with CAR T cell therapy is not well understood, and a spectrum of presentations is increasingly recognized. Here we report the case of a patient who developed transverse myelitis after receiving CAR T cell therapy.

A 28‐year old woman with refractory primary mediastinal B cell lymphoma was referred for standard of care (SOC) CAR T cell therapy after receiving R‐CHOP (rituximab, cyclophosphamide, doxorubicin, vincristine, and prednisone) chemotherapy, radiation, and GDP (gemcitabine, dexamethasone, and cisplatin) salvage chemotherapy. Due to tumor burden with extensive abdominal involvement, she received bridging therapy with pembrolizumab, dexamethasone, and radiation to the right kidney (see treatment summary, Figure 1A). During lymphodepletion, the patient developed high fevers of up to 40°C, with groundglass opacities on the CT thorax. No infectious etiology was determined; full septic workup, bronchoscopy, and COVID19 testing were negative.

**FIGURE 1:**
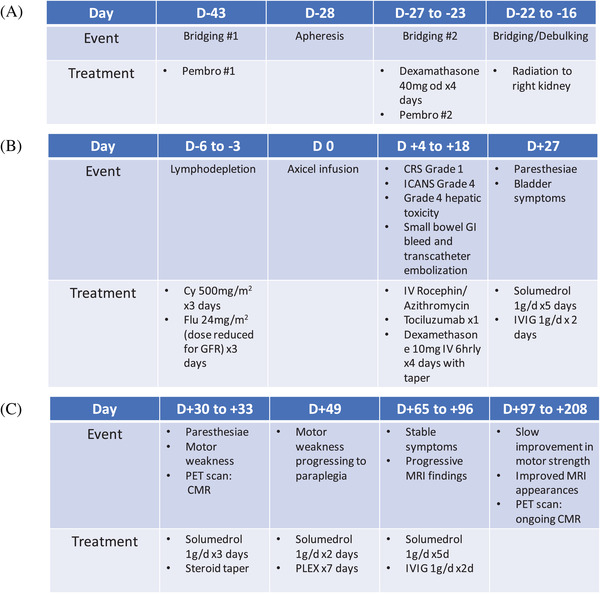
Treatment course. (A) Timeline pre‐bridging and lymphodepletion (B) Timeline lymphodepletion to D+27 (C) Timeline D+30 to D+208

The patient proceeded with Axi‐cel infusion. Baseline inflammatory markers on D 0 were elevated (Table 1, panel A). From D+1 to D+4, the patient continued to spike fevers >38°C, attributed to Grade 1 CRS (American society for transplantation and cellular therapy criteria). On D+4, the patient became nonverbal, unable to answer questions or follow commands, with myoclonic jerks but no focal motor or sensory symptoms. Her immune effector cell associated encephalopathy score was 0/10 (Grade 4 ICANS). Grade 4 hepatotoxicity (CTCAE v5.0) developed on D+5 (Figure 1B). There were no acute findings on brain MRI (magnetic resonance imaging), and an EEG showed generalized rhythmic delta activity with intermittent generalized periodic discharges; an extremely elevated protein level on lumbar puncture (LP) was consistent with neurotoxicity (Table 1, panel B). The patient received tocilizumab x 1 on D+4 and a dexamethasone taper to D+18 (Figure 1B). Fever and hepatotoxicity resolved from D+5; her neurological status returned to baseline on D+13.

**TABLE 1:** Summary of the patient workup for inflammatory and infectious causes

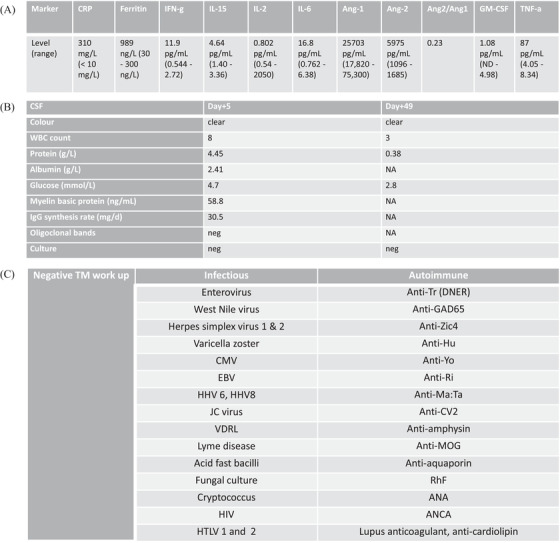

At routine outpatient follow‐up on D+27, the patient reported ascending paresthesiae to the chest area. Neurological examination showed hyperreflexia, diminished sensation to pinprick, vibration, and temperature in both lower extremities, impaired proprioception in the toes, and unsteady gait; there was no motor weakness.

Small bilateral subdural collections thought to be subacute in the setting of a chronically low platelet count were demonstrated on MRI brain, but, otherwise, there were no acute changes. MRI of the spine showed a subtle signal abnormality from T1–T7 within the dorsal central cord, concerning for transverse myelitis. A D+30 response assessment PET scan demonstrated a complete metabolic response from lymphoma (Figure 3).

The patient received treatment with steroids and intravenous immunoglobulin (IVIG) for presumed transverse myelitis but returned on D+33 with worsening ascending numbness, bilateral leg weakness, and urinary retention (Figure 1). On examination, there was a T4 sensory level to pinprick, bilateral leg weakness (4/5 in a pyramidal distribution), and extensor plantar responses. MRI of the spine showed an abnormal signal in the cord from T1–T7, in keeping with transverse myelitis (Figure 2). Pulsed IV steroid was given; the patient was discharged on a prednisone tapering schedule but returned on D+49 with paraplegia. Repeat LP was unremarkable (Table 1, panel B), and MRI of the spine demonstrated persistent and increased abnormal signal intensity in the central cord and posterior columns, unchanged in size. An extensive workup for autoimmune and infectious causes of transverse myelitis was negative (Table 1, panel C). The patient received further steroid therapy and plasma exchange (PLEX), with no significant improvement in symptoms. Follow‐up MRI (D+65) showed progression of myelitis with extension of signal change from C7 to T7 with enhancement. Further steroid therapy with a slow taper and IVIG were given.

**FIGURE 2:**
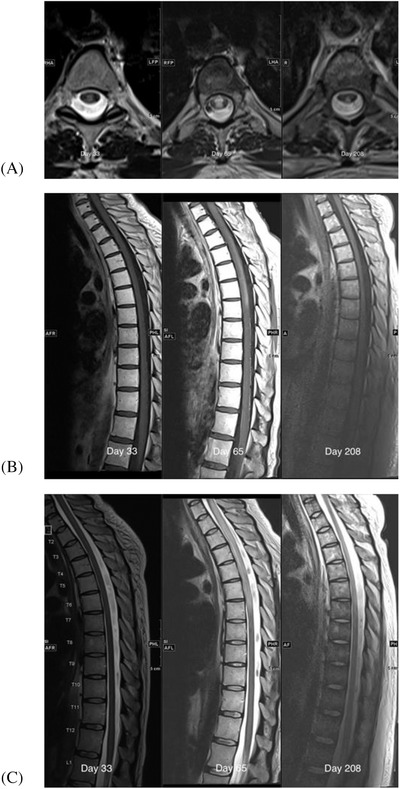
MRI images D+33, D+65 and D+208: (A) axial T2 weighted, (B) sagittal T1 weighted post‐gadolinium, and (C) sagittal T2 weighted images

**FIGURE 3:**
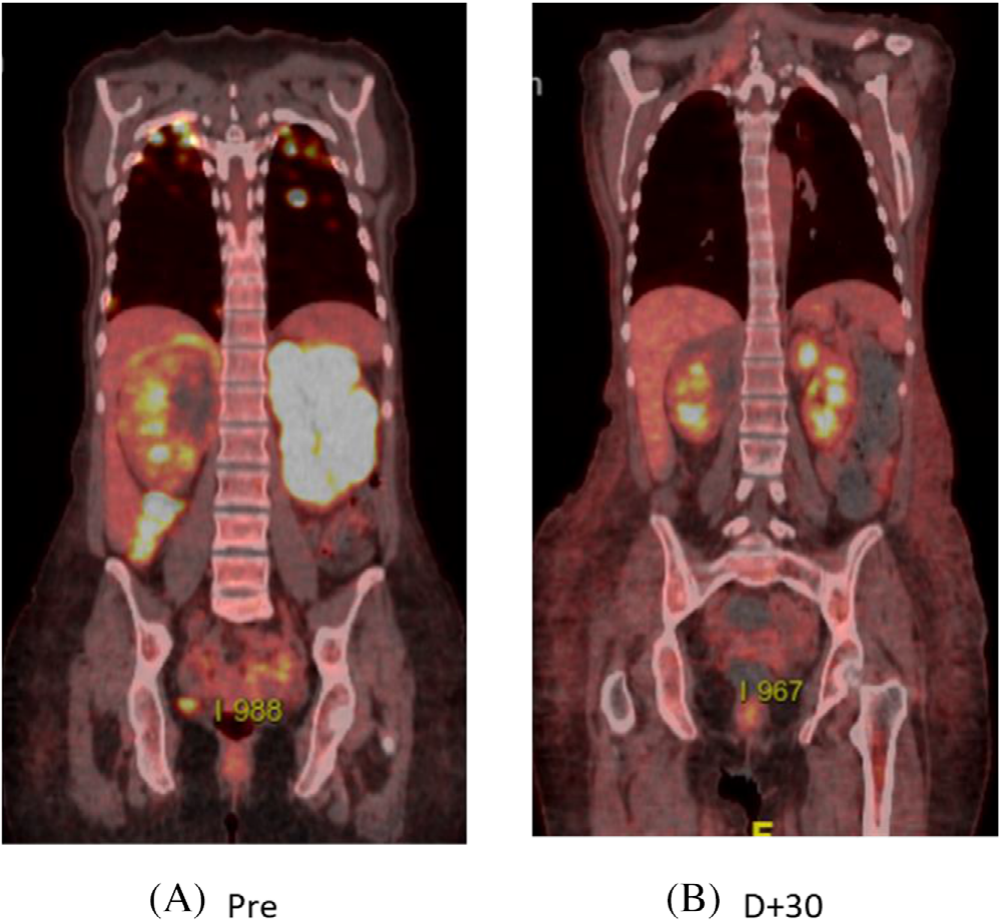
Positron emission tomography images: (A) prior to and (B) at D+30 post‐CAR T cell therapy

Over the next 4 months, the patient regained some motor strength in the lower limbs (2/5 hip flexion, 4/5 plantar flexion) and reported improvement in sensory symptoms (pinprick sensation to the level of the hips bilaterally). Follow‐up imaging of the spine on D+208 showed significant improvement in cord signal change from T1 ‐ T6 with resolution of enhancement (Figure 2). A positron emission tomography (PET) scan on D+104 showed ongoing complete metabolic remission from lymphoma (Deauville score 2).

We describe an unusual case of transverse myelitis after treatment with anti‐CD19 CAR T cell therapy, with a relapsing/remitting course over a 5‐month period before stabilization and improvement of symptoms. Treatment with CAR T cells was preceded by the administration of a checkpoint inhibitor targeting PD‐1 due to efficacy in primary mediastinal B cell lymphoma [12]. Checkpoint inhibitor treatment itself can be associated with neurological complications but can be combined with CAR T cell therapy with a manageable safety profile [13, 14, 15].

Neurotoxicity following CAR T cell therapy is common but usually self‐limited and reversible. Pivotal studies of CAR T cell therapy in lymphoma reported ICANS Grade ≥3 in 10–28% of patients, although grading schemes have differed [1, 2, 3]. A biphasic pattern has been described, with the first phase often occurring in the context of CRS, and a second delayed phase of presentation in approximately 10% of patients [4, 5, 6, 7]. Clinical manifestations of neurotoxicity can vary widely, ranging from headache to encephalopathy, seizures, and cerebral edema. Atypical presentations, including acute leukoencephalopathy and transverse myelitis, have also been reported [8, 16].

The pathophysiology driving neurotoxicity is not well understood. Several reports have implicated endothelial activation and blood–brain–barrier (BBB) disruption which may facilitate the influx of cytokines, recruitment of monocytes, and activation of macrophages, but elevated levels of the excitatory N‐methyl‐D‐aspartate receptor agonists glutamate and quinolinic acid have also been described [6, 9, 17].

Risk factors associated with the development of neurotoxicity include patient‐related factors such as younger age, higher tumor burden, and a history of early and/or high‐grade CRS, as well as product‐related characteristics such as CAR design and choice of lymphodepletion regimen [2, 5, 6]. Our patient presented with multiple risk factors including young age, extensive tumor burden at relapse, and she received a CD28‐costimulated CAR T cell product [10]. She had a particularly high baseline ANG2 (Table 1, panel A), known to be associated with increased endothelial cell activation and posited to represent BBB permeability [9]. An association between baseline IL‐6, ANG2, ANG2/ANG1, and ferritin, with subsequent severe neurotoxicity after CAR T cell therapy has been described [11]. Indeed, this patient developed evidence of severe BBB permeability after CAR T infusion with a high cerebrospinal fluid (CSF) protein level on D+5.

One limitation of this case report is that in the SOC setting CAR T cells are not routinely quantified or phenotyped, and, therefore, we could not determine if there were any CAR T cell characteristics that differed in this case, and neither was it possible to determine the presence of CAR T cells in the CSF. The patients who developed leukoencephalopathy in the case report from ZUMA‐1, for example, were found to have a massive expansion of peripheral CAR T cells and prominent features of BBB disruption [8].

Interestingly, it is also unclear whether any of the treatments pursued (pulsed corticosteroids, PLEX, IVIG) changed the disease course or whether time alone leads to disease improvement in such cases. Rituximab was not administered as CD19 CAR T cell therapy strongly depletes B cells, and additional B cell depletion was considered unlikely to be of benefit.

As a relatively new technology, rare and unexpected side effects may occur in patients receiving CAR T cell therapy. Reporting and collection of such cases will help to increase awareness of rare presentations and advance the expertise in managing these challenging cases.

## CONFLICT OF INTEREST

SS, SM, JAS, KR, NF, DW: No disclosures. RF: Research funding from Novartis and Kite. MLD: Research funding from Celgene, Novartis, Kite, and Atara; other financial support from Novartis, Precision Biosciences, Celyad, Bellicum, and GlaxoSmithKline; stock options from Precision Biosciences, Adaptive Biotechnologies, and Anixa Biosciences. FLL: Scientific advisory role with Kite, a Gilead Company, Novartis, Celgene/Bristol‐Myers Squibb, GammaDelta Therapeutics, Wugen, Amgen, Calibr, and Allogene; consultant with grant options for Cellular Biomedicine Group, Inc.; research support from Kite, a Gilead Company. MDJ: Consultancy/advisory role for Kite/Gilead, Novartis, Takeda, and BMS. JGK: Consultant or advisory role for Abbvie, BMS, Gilead, Karyopharm, Merck, Roche, Seattle Genetics; honoraria from Amgen, Antengene, Astra Zeneca, BMS, Gilead, Incyte, Janssen, Karyopharm, Merck, Novartis, Pfizer, Roche, Seattle Genetics, TG Therapeutics; research funding from Canadian Cancer Society, Leukemia and Lymphoma Society Canada, Princess Margaret Cancer Foundation, Janssen, Roche, Astra Zeneca; other remuneration from Karyopharm (DSMB).

## AUTHOR CONTRIBUTIONS

KR and RF performed cytokine and data analyses and reviewed the manuscript; JAS, MLD, NF, and FLL critically reviewed and edited the manuscript; SS, MDJ, DW, and JGK analyzed data and wrote and edited the manuscript.
